# Developing New Health Material: The Utilization of Spray Drying Technology on Avocado (*Persea Americana* Mill.) Seed Powder

**DOI:** 10.3390/foods9020139

**Published:** 2020-01-30

**Authors:** Karen Alissa, Yu-Chi Hung, Chih Yao Hou, GiGi Chin Wen Lim, Jhih-Ying Ciou

**Affiliations:** 1 Department of Food Science, Tunghai University, Taichung 407, Taiwan; karen.alissa23@gmail.com (K.A.); gg0680@gmail.com (G.C.W.L.); 2 Department of Food Technology, i3L–Indonesia International Institute for Life Sciences, Jakarta Timur 13210, Indonesia; 3 Department of Seafood Science, National Kaohsiung University of Science and Technology, Kaohsiung 811, Taiwan; a0922218820@gmail.com (Y.-C.H.); chihyaohou@gmail.com (C.Y.H.)

**Keywords:** avocado seed, spray drying, avocado seed powder, inlet temperature, feed flow rate, maltodextrin

## Abstract

Avocado (*Persea Americana* Mill.) generates byproducts, especially the avocado seeds. Hence, the aim of this study was to investigate the potential utilization of avocado seed as a very important, high phenolic content, climacteric fruit with unique characteristics and high nutritional properties. As such, theantioxidative test is conducted, then spray drying is used to produce avocado seed powder. The objective of this study was to develop an avocado seed powder using the spray drying technique by investigating the solution stability with different avocado seed extract concentrations, and to determine the physical properties of spray dried avocado powder that consists of powder yield, moisture, water activity, solubility, and color. The avocado seed extract was mixed with maltodextrin and water and homogenized for 10 min at 8000 rpm. The avocado seed solution was then spray dried with different inlet temperatures and feed flow rates. The spray dried avocado seed powder was analyzed for its yield, moisture content, water activity, solubility, and color. It was reported that the solution with the least avocado extract concentration (10 g) had the best stability in terms of presence of solute particles and color. The avocado seed powder obtained from this experiment had yield ranges from 24.46–35.47%, moisture content ranges from 7.18–7.96%, water activity ranges from 0.27–0.34, solubility ranges from 55.50–79.67 seconds, L* value ranges from 38.38–41.05, a* value ranges from 6.20–7.25, and b* value ranges from 13.33–15.17. In addition, increasing inlet temperature resulted in an increase in powder yield, solubility, a* value, and b*value, as well as a decrease in moisture, water activity, and L* value. Meanwhile, increasing the feed flow rate resulted in an increase in powder yield, moisture, water activity, and all L*, a*, b* values, as well as a decrease in solubility. In conclusion, spray drying technology is able to develop avocado seed powder.

## 1. Introduction

The avocado (*Persea Americana* Mill.) is a climacteric fruit originating from Central America, specifically from Mexico, Guatemala, and West Indie [1,2]. Then avocado has some unique characteristics, such as its pear-like shape, green rough skin, and a smooth pulp texture. The avocado possesses high nutritional content. It is specifically high in monounsaturated fat—which is associated with the reduction of cholesterol [3]—as well as vitamins, minerals, and phytochemical compounds. The consumption of avocado is often related to immune system improvements and protection against oxidative damage in the organism [2]. This high nutritional profile of avocado makes avocado an ideal complementary food that could provide adequate nutrients and energy for the human body.

Unfortunately, the processing of avocado still generates byproducts, especially the avocado seeds, as most industrial applications only utilize the pulp. Although the seeds represent a considerable percentage of the total fruit, they are often under-utilized, discarded, and thus become waste [4]. According to Araújo et al. [3], the production of avocadoes reaches 5 million tons per year, which makes the avocado the fourth most important tropical fruit worldwide [3]. In Indonesia, there was an increase of avocado imports from 7401 to 8251 kg in 2016. In addition, the production of avocadoes kept increasing from 2010 to 2017, reaching 363,000 tons in 2017. This indicates that there is a large increase of avocado production and consumption, which also leads to an increase in the waste of avocado seeds. This phenomenon could be a problem because the waste could cause ecological problems and economic losses due to the high cost of transporting them to disposal areas [5]. Therefore, this study aims to investigate the potential utilization of these byproducts; this is very important since it could increase the value of the avocado seeds and reduce waste at the same time.

Moreover, avocado seed is known to possess many health benefits since it contains high phytochemical compounds such as phytosterols, triterpenes, fatty acids, furanoid acids, flavonol dimers, proanthocyanidins, and abscisic acid [5]. According to Dabas et al. [6], avocado seeds could treat hypercholesterolemia, hypertension, and inflammatory conditions [6]. The seed extract could also treat diabetes by decreasing blood glucose [7]. Avocado seeds also possess insecticidal, fungicidal, and antimicrobial activities [8]. Moreover, Geissman & Dittmar (1965) also claimed that avocado seed extracts are rich in polyphenolic compounds that exhibit antioxidant properties, including (+)-catechin, (−)-epicatechin, and proanthocyanidin compounds [9].

The utilization of avocado seeds could be enabled by using spray drying, which converts the avocado seed extract into avocado seed powder. Spray drying is a widely used technique used in food industries to convert liquids into solids in order to increase the shelf life and stability of the end product [10]. It works by removing the moisture content and thereby water activity of the food products, hence decreasing the enzymatic reaction, inhibiting microbial growth, and reducing spoilage. Spray drying has been used in various types of food products, including fruits, such as watermelons [11], tomatoes [12], pineapples [13], blackberries [14], pomegranates [15], bananas [10], and even avocadoes themselves [2]. However, studies regarding the spray drying of avocado seed extract are still limited. Spray drying of avocado seed extract by using maltodextrin as a carrier is expected to reduce the waste of the avocado seed by transforming it into avocado seed powder, to increase its value by turning it from waste into food product, and to allow for the potential to incorporate the powder into various products such as instant soups and beverage products.

## 2. Materials and Methods 

### 2.1. Sample Preparation

The avocado seed extract was obtained from avocado seed as described in Section 2.2.1. It had a light solid orange color with a slightly thick texture. Prior to use, the avocado extract was stored in a freezer (−18 °C) to maintain its quality and prevent spoilage.

### 2.2. Solution Preparation and Stability Test

#### 2.2.1. Solution Preparation

The avocado seed was evenly sliced and homogenized. Then, the puree was mixed with water at the ratio of 1:5, then ultrasonic shock was used to dissolve phenolic compounds. The sample was then ready after the mixture was vacuum filtered, and could then be stored in a fridge after it was concentrated.

#### 2.2.2. Brix and pH Value

The pH value and sugar content of the ultra-sounded and vacuum-filtered liquid was determined by a pH meter (Suntex, Taiwan) and Brix meter (Atago, Tokyo, Japan), with triplicates and average values calculated.

#### 2.2.3. Total Phenolic Content

Quantities of 1 mL of sample, 0.5 mL of Folin-Ciocalteus reagent, and 3 mL of 20% Na_2_CO_3_ were mixed and sat for 15 min for the reaction. Then, 5 mL of deionized distilled water was added before testing. A spectrophotometer with wavelength of 750 nm was used to determine the absorbance (gallic acid used as standard) and the unit was recorded as mg/mL.

#### 2.2.4. Reducing Power

The reducing power was used to test the preventive antioxidant function. Although it did not have a direct relationship to total phenolic determination, it could be assisted by DPPH and ABTS. One milliliter of avocado seed extracts of each concentration were added to 1 mL of 200 mM phosphate buffer solution (pH 6.6) and mixed with 1 mL of Potassium Ferricy Anode. The mixtures were then reacted in a water bath at 50 °C for 20 min. After rapid cooling, 1 mL of 10% Trichloroacetic acid (TCA) solution (prepared with 95% ethanol) was added. After centrifuging the sample at 3000 rpm for 10 min, 100 μL of the supernatant was taken and added to 100 μL DW, 100 μL of 0.1% Ferric chloride (FeCl_3_·6H_2_O) solution, and 3.5% hydrochloric acid solution, mixed well, and left to stand for 10 min. The enzyme absorbance (ELISA reader) was then used to measure the absorbance of the samples at 700 nm [16]. The higher the absorbance was, the stronger the reducing ability was. The iron reducing ability potential determination method was used to evaluate the antioxidant activity of avocado seed extract, in which the ability of Fe^3+^/Ferricyanide complex to reduce to ferrous (Fe^2+^) form is measured. The chemical principle is based on the electron transfer reaction, in which potassium ferricyanide is the oxidant, and the antioxidant acts as a reducing agent by supplying hydrogen atoms to the iron complex and will destroy the free radical chain reaction [17].

#### 2.2.5. ABTS Method(2,2′-azino-bis[3-ethylbenzothiazoline-6-sulfonate])

A quantity of 2.45 mM of solution (7 mM ABTS (2,2-azinobis [3-ethylbenzothiazo line-6-sulfonic acid]) and potassium persulfate) was placed to react for 12–16 h under dark, room temperature conditions. Stabilized ABTS radical cation solution was diluted with PBS to absorbance of 0.70 (±0.02) at 730 nm. Then, 1 mL of diluted solution was added with 10 μL of the different concentrations of samples and reacted for a minute, then tested under 730 nm [18].

#### 2.2.6. DPPH Method

The antioxidant activity of avocado seed concentrate was measured using the method developed by Alara et al., with minor modifications [19]. For this, 200 μL of each concentration of methanol extract was added to 200 μL of DPPH (0.1 mM) solution. The mixture was shaken and left in a dark drawer at room temperature for 30 min. The absorbance of the sample was then measured using an ELISA reader at a wavelength of 517 nm. Methanol was used as a blank and the equation was used to calculate the scavenging activity for DPPH. In order to find the antioxidant effect as a percentage of the concentration of avocado seed extract, the semi-inhibitory concentration (IC_50_) of the sample was also calculated.

DPPH radical scavenging activity (%) = ((A_control_ – A_sample_)/A_control_) × 100, (1)

where A_control_ represents the absorbance of DPPH and methanol solution without the sample, and A_sample_ represents the mixture of sample (avocado seed extract) and DPPH solution.

### 2.3. Spray Drying

The chosen formula listed in Table 1 was prepared to be spray dried. The spray drying process was carried out in a laboratory spray dryer, Yamato Pulvis GB 22 model, with inlet temperature and feed flow rate as the independent variables ranging from 160 to 200 °C and 20 to 25 mL/min, respectively. The formula or combination between those two independent variables was generated using Design-Expert 9 Software (version 9, Stat-Ease Inc., Minneapolis, MN, USA). based on the experimental design described in Section 2.4 below. The drying air flow rate was kept constant at 0.45 m^3^/min while the air pressure was kept between the range of 0.10 to 0.30 MPa. The powder obtained from the spray drying process was put in a sealed plastic bag and stored at desiccator cabinet containing silica gel until further analysis.

### 2.4. Regression Model and Statistical Analysis 

This study utilizes response surface methodology (RSM) to investigate the effects of process variables on various responses. RSM is an optimization of analytical procedures using the multivariate statistical technique [20]. Traditionally, the analysis was done using one-variable-at-a-time method by monitoring the influence of one factor at a time on an experimental response, in which only one parameter was changed while the other was kept at a constant level. However, this method could not measure the interactions among the variables and increased the number of experiments necessary to conduct the research, which lead to an increase in the consumption of materials, time, and expenses [21]. By using RSM, it allows the simultaneous optimization of a response that is influenced by several independent variables. There are two terms used for the RSM method: Factors (independent variables) and levels (different values of the variables) [20,21].

In this study, RSM was used to investigate the effect of spray drying conditions on various responses, such as yield (%), moisture content (%), water activity, solubility (sec), and color. A central composite design (CCD) of two factors and 5 levels was utilized to design the experiment. The two factors were the inlet temperature (160–200 °C) and feed flow rate (20–25 mL/min). Five levels of each variable were chosen for the trials, including the central point and two axial points [22]. CCD is an effective method to provide information on experimental variable effects [23]. The data were analyzed using RSM by Design-Expert 9 Software.

### 2.5. Avocado Seed Powder Analysis

#### 2.5.1. Powder Yield

The powder yield was calculated as the ratio of the amount of powder collected after spray drying to the initial amount of solids in the feed solution.

#### 2.5.2. Moisture Analysis

The avocado seed powder sample (2 g) was placed in A&D ML-50 moisture analyzer, in which the instrument automatically measured the moisture content of the powder. The measurement was done in triplicate.

#### 2.5.3. Water Activity Analysis 

The avocado seed powder sample (2 g) was placed in an AquaLab Lite water activity meter at 25 °C until the instrument finished the measurement. The measurement was done in triplicate.

#### 2.5.4. Solubility Analysis

Avocado seed powder (1 g) was weighed and 300 mL of distilled water was heated until it reached 70 °C. Both were mixed and the mixture was placed on a hot plate and stirred with a constant speed using a magnetic stirrer. The time used to completely dissolve the avocado powder was recorded and the measurement was done in triplicate [10].

#### 2.5.5. Color Analysis

The color properties of the avocado powder were determined using 3nh NR 110 precision colorimeter in terms of L* (lightness), a* (redness, greenness), and b* value (yellowness, blueness). The measurement was done in triplicate. L* represents the lightness to darkness (0–100), a* represents redness (positive) and greenness (negative), while b* represents yellowness (positive) and blueness (negative).

#### 2.5.6. Scanning Electron Microscopy (SEM)

Avocado seed waste was collected and freeze-dried for SEM analysis. The morphological properties of the seed powder were also observed through SEM (Quanta 200, FEI, Hillsboro, OR, USA), vacuuming to 10^−6^ mbar, and taking 60 s to take a photo.

### 2.6. Statistical Analysis

All the data obtained were recorded and calculated for the mean and standard deviation. For the solution stability analysis, the variance was statistically analyzed using one-way ANOVA and post-hoc Turkey test. As for the avocado seed powder analysis, RSM with two factors central composite design (CCD) was employed to investigate the interaction between the variables via Design-Expert 9 statistical package. The relationship between the response and two independent variables (inlet temperature and flow rate) was evaluated using second-degree polynomial model as shown below.

(2) $$Y_{1}=\;\beta_{0}+\;\beta_{1}X_{1}+\;\beta_{2}X_{2}+\;\beta_{11}X_{1}{}^{2}+\;\beta_{22}X_{2}{}^{2}+\;\beta_{12}X_{1}X_{2}+\;\varepsilon .$$

*Y*_1_ was the response, *X*_1_ is the inlet temperature, *X*_2_ is the flow rate, *β*_0_ is the intercept, *β*_1_ and *β*_2_ are the linear coefficients, *β*_11_ and *β*_22_ are the quadratic coefficients, and *β*_12_ is the interaction coefficient. The analysis of variance (ANOVA) was generated for each response functions and the three-dimensional (3D) surface plots were used to study the interactive effect of the independent variables on the responses. All tests were done with significance level (α) of 0.05.

## 3. Results and Discussion

### 3.1. Solution Stability

Table 2 represents the results of sugar content and pH value of avocado seed extract. The sugar content of the avocado seed extract was 1.23 ± 0.047 °Brix. The pH value of avocado seed extract was 5.76 ± 0.024, which is a weak acidic substance. The values are taken from average of triplicates measurements.

Previous studies have showed that phenol is one of the major plant compounds with antioxidant activity [24]. It was reported that the antioxidant activity of phenolic and polyphenolic compounds was mainly due to their redox properties, and they play an important role in absorbing and neutralizing free radicals, quenching singlet and triplet oxygen, or decomposing peroxides [24]. In this study, total phenolic content was determined using the Folin-Ciocalteau assay and the results are expressed as gallic acid equivalents. The phenol content of avocado seed extract was equivalent to 367.13 ± 0.26 mg/g of gallic acid (Table 2). By comparison, avocado seed extract has 2–3 times the phenolic content of camellia seed extract [25]. According to research, it appears that the phenolic compounds of avocado seeds contain catechins, flavonoids, tannins, and phenolic compounds, which are also the main antioxidant sources of avocado seeds [8]. The results showed a positive correlation between the oil’s antioxidant activity and its total phenol content.

The methods which are commonly used for antioxidants are DPPH and ABTS; hence, they can be used to evaluate the antioxidant capacity of avocado seed extract at all tested concentrations. The DPPH radical inhibition test is a widely used and relatively easy method to evaluate antioxidant activity. DPPH is a stable free radical that produces a purple solution in ethanol. It is reduced and decolorized in the presence of antioxidant molecules and is often used to evaluate free radical scavenging activities of antioxidants, such as natural and synthetic pure compounds and plant extracts [24]. The ability to scavenge free radicals can be demonstrated by the degree of discoloration of the sample or extract. The DPPH removal efficiency of avocado seed extract is shown in Table 2. In this research, the degree of antioxidant activity of avocado seed extract was known from the 50% inhibitory concentration (IC_50_). The IC_50_, of avocado seed extract was 65.28 ± 0.12 μg/mL (Table 2). This value shows that avocado seed extracts have good antioxidant activity, thus, are known for both the antioxidant activity capacity and biological activity. In addition, avocado seed extract also shows some compounds with anti-inflammatory activity [26]. This study also used ABTS analysis to check the antioxidant activity of avocado seed extract. It can be seen from Table 2 that the ABTS analysis (IC_50_ = 2.767 ± 0.08 μg/mL) has a stronger antioxidant capacity for avocado seed extract than the DPPH analysis (IC_50_ = 65.28 ± 0.12 μg/mL). Lower IC_50_ value means higher antioxidant activity. Both DPPH and ABTS compounds have proton free radicals and are significantly reduced when exposed to proton free radical scavengers. Most plant compounds have better antioxidant activity against ABTS free radicals than DPPH free radicals [19]. This is because the ABTS assay is more sensitive in identifying antioxidant activity, which makes the kinetic response faster, resulting in higher antioxidant activity. The results are shown in Table 2. The absorbance of the sample was measured at 700 nm by using an enzyme immunoassay analyzer (ELISA reader). The higher the absorbance is, the stronger the reducing ability is. The OD value of the reducing power measurement is 1.70 ± 0.06.

### 3.2. Regression Model and Statistical Analysis

The relationships between both inlet temperature (*X*_1_) and flow rate (*X*_2_), and powder yield (*Y*_1_), moisture content (*Y*_2_), water activity (*Y*_3_), solubility (*Y*_4_), L* value (*Y*_5_), a* value (*Y*_6_), and b* value (*Y*_7_) were studied. Based on Table 3, the fit models in the coded factors are as follows:

(3) $$Y_{1}=\;26.07+\;4.06X_{1}+\;2.07X_{2}+\;1.78X_{1}{}^{2}-1.53X_{2}{}^{2}+\;0.98X_{1}X_{2},$$

(4) $$Y_{2}=\;7.56\;-0.25X_{1}+\;0.05X_{2},$$

(5) $$Y_{3}=\;0.32-\;0.015X_{1}-\;1.464E-003X_{2},$$

(6) $$Y_{4}=\;66-6.81X_{1}+\;1.21X_{2},$$

(7) $$Y_{5}=\;40.52-\;0.81X_{1}+\;0.24X_{2}-\;0.39X_{1}{}^{2}-\;0.22X_{2}{}^{2}+\;0.075X_{1}X_{2},$$

(8) $$Y_{6}=\;7.08-\;0.066X_{1}-\;0.11X_{2}-\;0.21X_{1}{}^{2}-\;0.21X_{2}{}^{2}-\;0.11X_{1}X_{2},$$

(9) $$Y_{7}=\;14.54+\;0.02X_{1}-\;0.21X_{2}-\;0.39X_{1}{}^{2}-\;0.11X_{2}{}^{2}-0.17X_{1}X_{2}.$$

### 3.3. Powder Yield

**Figure 1:**
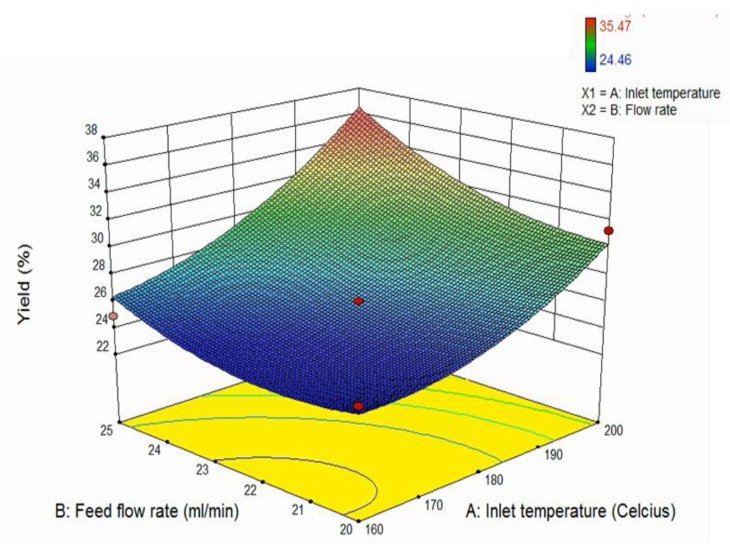
Three-dimensional surface plot of the effect of inlet temperature and feed flow rate to the powder yield.

Figure 1 represents the change of powder yield with varying inlet temperature and flow rate, which has a quadratic model. From the graph, it can be seen that the powder yield is increased with increasing inlet temperature and flow rate (*p* < 0.05). As seen in the results, the powder yield obtained from this experiment ranged from 24.46 to 35.47%. These values are considered low, as an operation is considered successful when it has at least 50% of yield [27]. However, these values are still in range with other works, such as avocado pulp powder (22.6–44.8%), jussara pulp (33.88–76.55%), and acai (34.39–55.66%) [2,22,28]. The main reason for this phenomenon to occur is due to the powder stickiness on the drying chamber [27]. During the process of spray drying, it could be observed that some of the powder stuck on the wall of the drying chamber. Powder stickiness can be caused by many factors, including the drying temperature being above the glass transition temperature of the avocado seed. When the temperature used is above the glass transition temperature, it causes the transformation of the surface of the material into viscoelastic rubbery state, which causes them to stick to the drying chamber [27]. The other possible reason that could cause stickiness is that the carrier agents used in this experiment is not enough. Maltodextrin, which acts as the carrier agent, is used to increase the glass transition temperature of the material [27,29]. However, there was only a formula of maltodextrin concentration used in the study, which maltodextrin concentration could be used to hypothesize was insufficient. For future studies, using higher concentrations of maltodextrin could be done to improve the product yield. It was proven by previous studies conducted by Vardin & Yasar (2011), Fazaeli et al. (2012), Shishir et al. (2014), and Wong, Teoh, and Putri (2017) on pomegranate, black mulberry juice, pink guava powder, and banana powder, respectively, in which increasing the maltodextrin concentration resulted in an increase in product yield [10,30,31,32].

Figure 1 shows a significant (*p* < 0.05) increase of powder yield as the inlet temperature increases. This agrees with the literature, in which increasing inlet temperatures lead to greater efficiency of heat and mass transfer [27,29]. The result from this experiment is also in accordance with previous studies on avocado pulp, banana, and black mulberry juice [2,10,31]. On the other hand, it was also shown that there was a significant increase of powder yield as the feed flow rate increased. However, this result from this experiment contradicts the literature, in which feed flow rate should have a negative effect on process yield [27]. This is related to the increase in the droplet size at the beginning of the drying, which leads to larger surface area that causes slower heat and mass transfer. In addition, a higher feed flow rate could also cause dripping inside the drying chamber [27]. This contradicting result could be related to the systematic error of the spray drying machine used in this experiment. During the spray drying process, it could be observed that the powder only stuck on a specific area on the drying wall chamber and not on the other areas. This stickiness was also observed only during the last few minutes of the spray drying (not from the beginning of the process). This could be due to some material that stuck inside of the nozzle, causing the nozzle to not properly spray the solution. As mentioned before, avocado seed extract contains high amounts of carbohydrates or sugars. As the spray drying process continues, the solution is exposed to high temperatures. Some of the sugar inside the solution started to melt and stick to the nozzle, which blocked the tube inside the nozzle where the liquid passed through. Therefore, instead of spraying the liquid in a straight downward direction, the nozzle sprayed in a diagonal direction, facing only one specific area of the wall. This caused the powder to stick to the drying wall chamber and eventually affected the yield. Another possible reason is the dripping problem, as a result of the collision of the semi-wet particles on the wall [33]. This could be related to the different spray drying machine and different flow rate range used compared to other studies. In this study, the spray drying machine used was a lab scale machine, which is smaller than the industrial scale spray drying machine. The size of the machine could influence the obtained powder, especially the yield. Since the spray drying machine was small, the atomizer and the chamber were also smaller, meaning that there was a higher chance for the powder to hit the liquid solution.

### 3.4. Moisture Content

**Figure 2:**
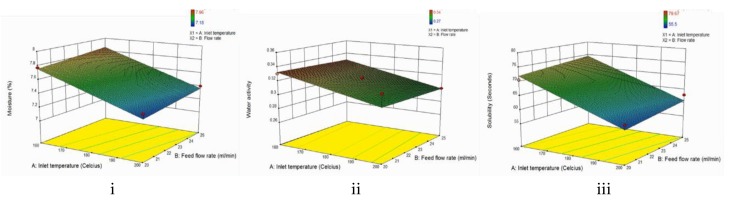
Three-dimensional surface plot of the effect of inlet temperature and feed flow rate to the (**i**) moisture content, (**ii**) water activity, (**iii**) solubility of powder.

Figure 2i represents the change of moisture content with varying inlet temperature and flow rate. From the graph, it can be seen that both independent variables have a linear effect on the moisture content. The moisture content of the powder is decreased with increasing inlet temperature but increased with increasing flow rate (*p* < 0.05). As seen in the results, the moisture content of the powder obtained from this experiment ranged from 7.18 to 7.96%. According to Tontul and Topuz (2017), the moisture content of a spray dried powder should be lower than 5% to be classified as microbiologically safe and stable during storage (to prevent caking) [27]. The moisture content obtained is considered high in this study. However, these values are still in range with other studies by Dantas et al. (2018) on avocado pulp and Goula and Adamopoulos (2008) on tomato pulp [2,12]. This phenomenon might be caused by the high macromolecule content of avocado seeds [34]. In macromolecules, such as proteins or carbohydrate, hydrogen bonds can be formed that cause water molecules to be built in the structure of the macromolecules. Water molecules refer to the structure of water when it is immobilezed in marcomolecule structure; hydration water is where water molecules reorient between bonding [35]. Since the macromolecules content of avocado seed is quite high, avocado seed is thus expected to have higher amounts of moisture. In addition, it could also be caused by the insufficient amount of carrier agent used for this experiment. This is because increasing the amount of carrier agent in the feed could decrease the moisture content of the powder [27]. This could be observed in some studies such as on black mulberry juice powder [31], pomegranate juice powder [30], banana powder [10], and blackberry powder [14].

The moisture content of the avocado seed powders significantly (*p* < 0.05) decreased with the increase in inlet temperature (Figure 2i). This is in accordance with the literature where higher inlet temperature and higher flow rate result in lower moisture content of the product [27]. This is because higher inlet temperature offers higher energy to the drying medium and increases the heat transfer. In addition, a higher inlet temperature also leads to the formation of powder with larger particle sizes and smaller surface areas. This is due to the faster drying rates that cause the early set up of the structure and do not allow the particle to shrink during the drying process [20]. As for the flow rate; a higher flow rate significantly (*p* < 0.05) increases the moisture content of the powder.This is also in accordance with the literature, where a higher flow rate results in higher moisture content. It is due to the higher flow rate that the size of the formed droplets increases and the contact time between droplets and drying air shortens [27]. Therefore, lower evaporation of the water in the droplet occurs because of less effective heat transfer. The same results could be observed in previous studies, including in pomegranate juice [15], watermelon [11], blackberry [14], passion fruit juice [36], acai [22], and avocado pulp [2].

### 3.5. Water Activity

Figure 2ii represents the change of water activity with varying inlet temperatures and flow rates. From the graph, it can be seen that both independent variables have a linear effect on the water activity. The water activity of the powder decreased with increasing inlet temperature but increased with increasing flow rate. However, both changes are considered as not significant (*p* > 0.05). As seen in the results, the water activity of the powder obtained from this experiment ranges from 0.27 to 0.34. These values are slightly higher compared to the standard of the water activity of the spray dried powder, which is 0.3 [27], but are still acceptable. It was stated that in this range of water activity, the powder could be indicated as microbiologically and chemically safe. There were no part of the reaction in which lipid oxidation, the browning reaction, enzyme activity, or microbiological growth occured. According to Adams and Moss (2008), almost all microbial activity is inhibited below 0.6, most molds below 0.7, yeasts below 0.8, and both gram positive and gram negative bacteria below 0.9 [37]. Meanwhile, oxidation will not occur at a water activity below 0.4, lipid oxidation is the lowest at water activity of 0.2 to 0.3, the browning reaction is maximum at 0.6, and most enzymes are inactivated below 0.85 [38]. Therefore, the spray dried powders obtained from this experiment are classified as safe and stable.

As seen in Figure 2ii, the water activity of the powder decreased with increasing temperature. However, the changes were not significant (*p* > 0.05). This is consistent with the literature, in which higher inlet temperatures lead to a higher evaporation rate, causing them to have lower water activity [2,27]. Similar results were found for avocado pulp [2], banana [10], and pomegranate [30]. On the contrary, the water activity increased with decreasing flow rate. Similar to the effect of inlet temperature, the changes were not significant (*p* > 0.05). This result is also consistent with the literature in which increasing feed flow rate has a positive effect on water activity, which is related to the explanation of the moisture content above [27]. Similar results were found for ginger extract [23] and carrot-celery juice [39].

### 3.6. Solubility

Figure 2iii represents the change of solubility of the powder with varying inlet temperatures and flow rates. As seen in the graph above, both independent variables have a linear effect on the solubility. Increasing the inlet temperature causes a decrease in the dissolution time, meaning that it increases the solubility of the powder (*p* < 0.05). However, a different effect was observed when increasing the feed flow rate. It could be seen that increasing the feed flow rate caused an increase in the dissolution time, meaning that the solubility of the powder was decreased (*p* > 0.05). In this study, the solubility was measured as the time needed for the powder to reconstitute or dissolve in the solvent. As seen in the results, the solubility of the powder ranged from 55.50 to 79.67 seconds. The values obtained from this study are in range with other studies on bananas (48.00 to 80.01 s) [10], pineapples (59.00 to 87.33 s) [40], and jussara pulp (78.81 to 92.75 s) [28]. There is no standard for solubility. However, higher solubility is more desired, especially if the powder is used as an additive in a food product, since it is easier to be reconstituted [27].

It can be seen on Figure 2iii that the solubility significantly (*p* < 0.05) increased with increasing inlet temperature. This is in accordance with the literature where higher inlet temperature will lead to a decrease in moisture content and an increase in the particle size of the powder [10,27]. When a particle is subjected to higher temperatures, rapid evaporation of moisture occurs at the surface of the particle. This phenomenon causes the formation of a crust on the surface that prevents the particle from shrinking during the drying process. During the dissolution process, these large particles will sink, making it easier for them to be dissolved in a solvent, thus a shorter time of reconstitution is required. Similar results can be observed in the study conducted by [10] on banana and [30] on pomegranate. Meanwhile, powder solubility decreased while flow rate increased, which is related to the high moisture content of the powder. Previous studies showed higher moisture content of powder lead to lower solubility [12,27]. Muzzafar and Kumar (2015) reported similar results in tamarind pulp and dates, respectively [41].

### 3.7. Color Analysis

**Figure 3:**
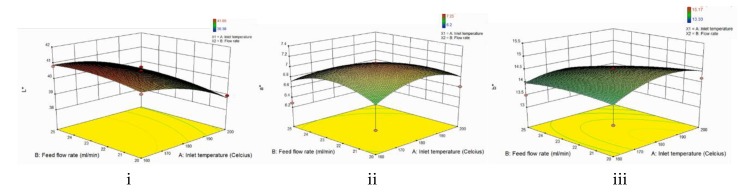
Three-dimensional surface plot of the effect of inlet temperature and feed flow rate to the color analysis of powder (**i**) L* value, (**ii**) a* value, (**iii**) b* value.

Figure 3 represents the change of color of the powder with varying inlet temperatures and flow rates. As seen on the graphs above, both independent variables had a quadratic effect on L*, a*, and b* values of the avocado powder. An increase in inlet temperature resulted in a decrease in the L* value (*p* < 0.05) and an increase in both a* and b* values (*p* > 0.05), meaning that it produced darker, redder, and more yellow powder. Meanwhile, an increase in flow rate resulted in an increase in all L* (*p* < 0.05), a* (*p* > 0.05), and b* values (*p* > 0.05), meaning that it produced lighter, redder, and more yellow powder. The results stated that the L*, a*, and b* values of the avocado seed spray dried powder ranges from 38.38 to 41.05, 6.20 to 7.25, and 13.33 to 15.17, respectively. These values vary depending on the raw material used, since different fruits have different colors. Therefore, there is no exact standard for the color analysis. Since the avocado extracts were orange in color, we expected to have both positive a* and b* values, which corresponds to the results obtained.

As seen in Figure 3, increasing the inlet temperature caused a decrease in the L* value but an increase in both a* and b* values. This means that as the inlet temperature increased, the powder had a darker, redder, and more yellow color. Moreover, only the decrease in the L* value was significant (*p* < 0.05), whereas the increase in both a* and b* values were either not significant (*p* > 0.05) or perceived the same. According to Ferrari, Germer, and de Aguirre (2011), lightness values were lower at a higher inlet temperature [14]. This is in accordance with the result from this experiment, as well as from previous studies on bananas [10], blackberries [14], and watermelons [11]. This phenomenon might occur due to the sugar content in the avocado seed that could lead to a browning reaction when exposed to high temperatures [27,39]. Another reason is due to the higher inlet temperature that causes more water (moisture) to be removed during the drying process, resulting in the concentration of the pigments [30]. This explains the reason for an increase in a* and b* values, as it means that the color of the spray dried powder is more concentrated (redder and more yellow). The same result was observed in the study conducted by Quek, Chok, and Swedlund (2007) on watermelons [11].

On the other hand, Figure 3 also shows that increasing the feed flow rate caused an increase in all the L*, a*, and b* values of the powder. This means that at a higher feed flow rate, the avocado seed powder has lighter, slightly redder, and more yellow color. However, increasing the flow rate only caused significant (*p* < 0.05) changes on the L* value, and not on the a* and b* values of the powder. Similar results were seen in the study on carrot-celery juice, where higher flow rate produced more yellow and orange colors [39]. This was due to the high feed flow rate that increases the volume to surface ratio of the powder and protected the color pigment.

### 3.8. Scanning Electron Microscopy (SEM)

**Figure 4:**
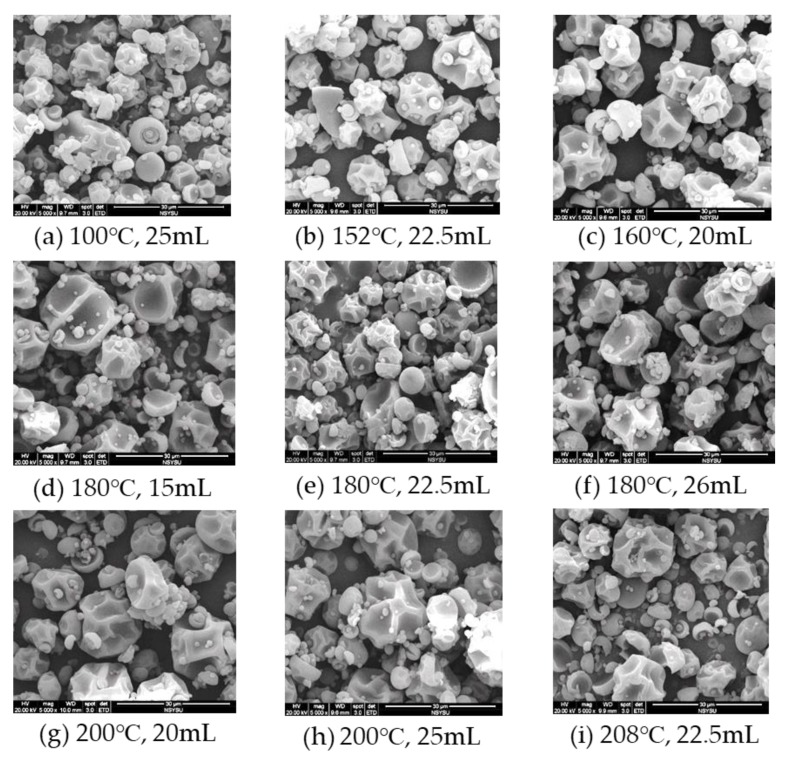
Scanning Electron Microscopy (SEM) analysis of powder with different conditions. (**a**) 100 °C, 25 mL, (**b**) 152 °C, 22.5 mL, (**c**) 160 °C, 20 mL, (**d**) 180 °C, 15 mL, (**e**) 180 °C, 22.5 mL, (**f**) 180 °C, 26 mL, (**g**) 200 °C, 20 mL, (**h**) 200 °C, 25 mL, (**i**) 208 °C, 22.5 mL.

Avocado seed extract was collected and freeze-dried for SEM analysis. The morphological properties of the seed powder were also observed through SEM (Quanta 200; FEI, Hillsboro, OR, USA), vacuuming to 10^−6^ mbar, and 60 s was taken to take a photo with a magnification of 5000×. Based on the SEM analysis results in Figure 4, the powder formed by spray drying resulted in inner moisture evaporating and adhering to solid, and due to the high temperature of granulation, the final powder appeared as sphere, and the surface shrunk. Therefore, the resultant the shrink phenomenon had a correlation with the spray dry temperature and affected the completeness of appearances. Below 100 °C, the diameter was about 5 μm and showed a smooth surface, but the particle size was unevenly spread compared to other conditions. At 152 °C and 160 °C, the diameter was 7 μm, where the surface was rough than 100 °C and particle size spread evenly. When the temperature increased to 180 °C, the particle size was evenly spread and tightly distributed, but the average diameter decreased to 3 μm. The highest temperature on this scanning was at 208 °C, thus the particle was not distributed evenly, and the particle size spread not equal.

## 4. Conclusions

In conclusion, spray drying technology is a viable method to develop avocado seed powder. Based on the results, it can be seen that the formulation with the least avocado extract concentration (10 g) was the most stable, as it has no presence of solute particles and the least color changes after 24 h storage at room temperature. The solution was spray dried with variable inlet temperatures and feed flow rates. It can be summarized that increasing the inlet temperature resulted in an increase in powder yield, solubility, a* values, and b*values, as well as a decrease in moisture, water activity, and L* values. Meanwhile, increasing the feed flow rate resulted in an increase in powder yield, moisture, water activity, and all L*, a*, b* values, as well as a decrease in solubility. However, according to the literature data, increasing the feed flow rate should result in a decrease in powder yield. This contradicting phenomenon might be due to the problem with the spray nozzle, the dripping problem, and the different spray drying machine used. In addition, both independent variables significantly changed all the responses above, except for the water activity, a*, and b* values. For future experiments, it is recommended that the pH of the solution is measured in order to have a clearer explanation of the stability of the mixture. In addition, it is also recommended to make maltodextrin concentrations as the independent variable, since the concentration of the carrier agents also influence the properties of the spray dried powder.

## Figures and Tables

**Table 1:** Spray drying conditions.

Trial *	Inlet Temperature (°C)	Feed Flow Rate (mL/min)
1	160	20
2	200	20
3	160	25
4	200	25
5	152	22.5
6	208	22.5
7	180	19
8	180	26
9	180	22.5
10	180	22.5
11	180	22.5
12	180	22.5
13	180	22.5

* The trials were generated using Design-Expert 9 Software.

**Table 2:** The sugar content and pH value of avocado seed extract.

	Sugar Content (°Brix)	pH Value	Total Phenolic Content (mg GAE/g)	Reducing Power (OD Value)	ABTS (IC_50_: μg/mL)	DPPH (IC_50_: μg/mL)
Avocado seed extract	1.23 ± 0.047	5.76 ± 0.024	367.13	1.70 ± 0.06	2.767	65.28

Averages are taken from triplicates measurements (*n* = 3). GAE = Gallic acid equivalent, OD value = Optical density value, ABTS = 2,2′-azino-bis(3-ethylbenzothiazoline-6-sulfonic acid), DPPH= 2,2-diphenyl-1-picrylhydrazyl.

**Table 3:** Coefficients of regression for the response equations for the independent variables.

Model	Yield (%)	Moisture (%)	Water Activity	Solubility (s)	Color		
					L*	a*	b*
	Quadratic	Linear	Linear	Linear	Quadratic		
β_0_	26.07	7.56	0.32	66	40.52	7.08	14.54
β_1_	4.06	−0.25	−0.015	−6.81	−0.81	−0.066	0.02
β_11_	1.78	-	-	-	−0.39	−0.21	−0.39
β_2_	2.07	0.050	−1.464 × 10^−3^	1.21	0.24	−0.11	−0.21
β_22_	−1.53	-	-	-	−0.22	−0.21	−0.11
β_12_	0.98	-	-	-	0.075	−0.11	−0.17
R_2_	0.96	0.97	0.51	0.93	0.978	0.44	0.48
